# Age-Related Changes in Micro Brain Characteristics Based on Relaxed Mean-Field Model

**DOI:** 10.3389/fnagi.2022.830529

**Published:** 2022-04-18

**Authors:** Ke Zhan, Yi Zheng, Yaqian Yang, Yi Zhen, Shaoting Tang, Zhiming Zheng

**Affiliations:** ^1^School of Mathematical Sciences, Beihang University, Beijing, China; ^2^Institute of Artificial Intelligence, Beihang University, Beijing, China; ^3^Key Laboratory of Mathematics, Informatics and Behavioral Semantics (LMIB), Beihang University, Beijing, China; ^4^State Key Laboratory of Software Development Environment (NLSDE), Beihang University, Beijing, China; ^5^Peng Cheng Laboratory, Shenzhen, China; ^6^Institute of Medical Artificial Intelligence, Binzhou Medical University, Yantai, China

**Keywords:** aging, micro brain characteristics, relaxed mean-field model, initial parameter sensitivity, recurrent connection strength

## Abstract

Brain health is an important research direction of neuroscience. In addition to the effects of diseases, we cannot ignore the negative effect of aging on brain health. There have been many studies on brain aging, but only a few have used dynamic models to analyze differences in micro brain characteristics in healthy people. In this article, we use the relaxed mean-field model (rMFM) to study the effects of normal aging. Two main parameters of this model are the recurrent connection strength and subcortical input strength. The sensitivity of the rMFM to the initial values of the parameters has not been fully discussed in previous research. We examine this issue through repeated numerical experiments and obtain a reasonable initial parameter range for this model. Differences in recurrent connection strength and subcortical input strength due to aging have also not been studied previously. We use statistical methods to find the regions of interest (ROIs) exhibiting significant differences between young and old groups. Further, we carry out a difference analysis on the process of change of these ROIs on a more detailed timescale. We find that even with the same final results, the trends of change in these ROIs are different. This shows that to develop possible methods to prevent or delay brain damage due to aging, more attention needs to be paid to the trends of change of different ROIs, not just the final results.

## 1. Introduction

Aging is an irreversible that all human beings must experience. All parts of the body are subject to aging, including the brain. Most somatic cells have a brief life, and new cells will replace them after apoptosis. However, things are different for neurons: they are longer-lived, and some even survive for the whole of an individual's lifetime. On the other hand, most neurons will not regenerate once they have become apoptotic. Therefore, aging will lead to serious damage to the brain structure, usually manifested as a decline in memory and cognition, and is accompanied by an increased risk of brain diseases such as Alzheimer's disease. Therefore, with the aim of preventing, or at least delaying, the aging of the brain, it is important not only to explore which regions of interest (ROIs) of the brain undergo changes in aging, but also to try to find out the processes involved.

Toward the end of the last century, researchers started to seriously study the aging of the brain. With the development of network neuroscience (Bullmore and Sporns, 2009; Bassett and Sporns, 2017), research on brain aging has developed in a number of directions. Chen (2019) summarized the current state of knowledge of the physiological basis of healthy aging and age-related neurodegeneration as revealed using functional magnetic resonance imaging (fMRI). More recently, Bi et al. (2021) combined MRI and positron emission tomography (PET) techniques in a study of 42 elderly normal-cognition subjects and elucidated the metabolic mechanism of the brain's structural connections and its relationship with normal aging. In another recent study, to examine the differences in structural connectivity and cognition underlying motor adaptation in visual-motor learning tasks, Wolpe et al. (2020) used structural MRI in over 300 subjects of different ages.

Many studies to date have been limited to either structural or functional connectivity alone, despite the fact that, especially in brain aging, these are closely interrelated (Honey et al., 2009). In many age-specific comparative studies, because of limitations of experimental design and the number of subjects, it is difficult to collect data from all age groups. It has also been pointed out that only using two cohorts for comparison cannot reflect the process of change from youth to advanced age (Wang et al., 2010).

In this study, we use the relaxed mean-field model (rMFM) (Wang et al., 2019) to simulate the dynamic process relating structural connectivity and functional connectivity. We then evaluate the model parameters through a comparison between the young group and the old group in all ROIs. The overall goal is to explore the process of change of brain differences with age, and thereby help prevent or delay brain aging in advance. First, we analyze the initial parameters of the rMFM model to explore the influence of different parameter initializations on the fitting results. We then establish whole-brain dynamic rMFMs in the young and old groups, respectively. After this, we compare microscale brain properties (the recurrent connection strength and subcortical input strength) of the two groups and identify the brain regions exhibiting significant differences. Finally, we use more samples from different age groups to explore the changes in these two micro brain characteristics with age.

## 2. Materials and Methods

### 2.1. Data

The data we used in this experiment were from the Nathan Kline Institute (NKI)/Rockland Sample public dataset. The dataset includes 196 subjects. Each subject received semi-structured diagnostic psychiatric interviews, and completed a battery of psychiatric, cognitive, and behavioral assessments. Then, after data acquisition by Siemens Trio 3T scanner, and preprocessing such as head movement correction, denoising, and thresholding, 188 ROIs were delineated according to the Craddock 200 atlas (Craddock et al., 2012), and the structural connectivity (SC) matrix and functional connectivity (FC) matrix obtained by diffusion tensor imaging (DTI) and fMRI. The dataset also included the subject ID, age, gender, name of ROIs (full and abbreviated), and the spatial position of ROIs in the brain. Some ROIs have the same names, and through mapping observation, we speculate that because these ROIs are in the same lobe and are very close in spatial location, the dataset author did not distinguish them by more specific naming. Therefore, we retained these same names, but dealt with them differently in model fitting, and we show their spatial location in the results.

The age range of subjects in the whole data set was [4, 85] (years). According to the aim of the study, we first placed subjects into a young group and an old group, depending on whether they were under the age of 20 or over 60, respectively, for finding ROIs where significant aging occurred. The data for subjects with ages in the range [20, 60] were used to characterize the changing trends in the aging process (see Table 1 for specific statistical information).

**Table 1:** Sample statistics.

	**Number**	**Age range**	**Age mean (±std)**	**Gender (M:F)**
DataSet	196	4–85	34.96 (± 20.04)	120:76
Young	53	4–20	13.79 (± 4.18)	29:24
Old	31	60–85	70.26 (± 7.20)	14:17

### 2.2. Relaxed Mean-Field Model

The whole-brain dynamic mean-field model (dMFM) (Deco et al., 2013) can be used to simulate neuronal activity through structural connection and is obtained by mean-field reduction of a detailed spiking neuronal network model within each brain region to the following set of nonlinear stochastic differential equations


$$\begin{array}{l} {\dot{S}}_{i}=-\frac{S_{i}}{\tau_{s}}+r(1-S_{i})H(x_{i})+\sigma v_{i}(t)\\ H(x_{i})=\frac{ax_{i}-b}{1-e^{-d(ax_{i}-b)}}\;\\ x_{i}=wJS_{i}+GJ\sum_{j}C_{ij}S_{j}+I \end{array}$$

where *x*_*i*_, *H*(*x*_*i*_), and *S*_*i*_ are the total input current, the population firing rate, and the average synaptic gating variable at the *i*-th cortical region, respectively. Recurrent connection strength *w*, subcortical input strength *I*, global scaling factor *G*, and neuronal noise σ are unknown parameters that are artificially given initial values and adjusted during model fitting, we will discuss the initial sensitivity of these parameters in the next subsection. Following previous work (Deco et al., 2013), the kinetic parameters for synaptic activity were set to be *r* = 0.641 and τ_*s*_ = 0.1*s*. Parameter values for the input-output function *H*(*x*_*i*_) were set to be *a* = 270*N*/*C*, *b* = 108*Hz*, and *d* = 0.154*s*. The value of synaptic coupling was set to be *J* = 0.2609*nA*. *v*_*i*_(*t*) is uncorrelated standard Gaussian noise, and the amplitude is controlled by σ.

The simulated neuronal activities *S*_*i*_ are fed to the Balloon-Windkessel hemodynamic model (Friston et al., 2003) to simulate the BOLD time series for each ROI. The specific process is, neuronal activity *S*_*i*_ in each ROI causes an increase in the vasodilatory signal *z*_*i*_, with an inflow *f*_*i*_ proportional to this signal, accompanied by changes in blood volume *v*_*i*_ and deoxyhemoglobin content *q*_*i*_. The equations concerning these neurophysiological processes are as follows


$$\begin{array}{l} {\dot{z}}_{i}=S_{i}-\kappa z_{i}-\gamma (f_{i}-1)\;\\ {\dot{f}}_{i}=z_{i}\;\\ \tau {\dot{v}}_{i}=f_{i}-v_{i}^{\frac{1}{\alpha }-1}\;\\ \tau {\dot{q}}_{i}=\frac{f_{i}}{\rho }\left[1-{\left(1-\rho \right)}^{\frac{1}{f_{i}}}\right]-q_{i}v_{i}^{\frac{1}{\alpha }-1} \end{array}$$

where the kinetic parameters rate of signal decay κ = 0.65*s*^−1^, rate of elimination γ = 0.41*s*^−1^. Hemodynamic transit time τ = 0.98*s*, and Grubb's exponent α = 0.32. ρ = 0.34 is the resting oxygen extraction fraction. When *q*_*i*_ and *v*_*i*_ are obtained, the BOLD time series is calculated by the following equation (Stephan et al., 2007; Heinzle et al., 2016).


$$\begin{array}{l} BOLD_{i}=V_{0}\left[k_{1}\left(1-q_{i}\right)+k_{2}\left(1-\frac{q_{i}}{v_{i}}\right)+k_{3}\left(1-v_{i}\right)\right] \end{array}$$

where *V*_0_ = 0.02 is the resting blood volume fraction and the equations for *k*_1_, *k*_2_, *k*_3_ are as follows


$$\begin{array}{l} k_{1}=4.3\vartheta_{0}\rho TE\;\\ k_{2}=\varepsilon r_{0}\rho TE\;\\ k_{3}=1-\varepsilon \end{array}$$

In the NKI dataset, the magnetic field strength *B*_0_ = 3*T*. At this time, the frequency offset of the outer surface of magnetized vessel ϑ_0_ = 28.265*B*_0_, the intravascular relaxation rate *r*_0_ = 110*Hz*, and the ratio between intravascular and extravascular MR signal is ε = 0.47. The echo time *TE* = 30*ms* in the NKI dataset.

We used a modified version of the MFM here, namely, the relaxed mean-field model (rMFM). In developing this model, Wang et al. (2019) relaxed the global parameters *w* and *I*, and optimized them into local parameters, so that each ROI has a pair of *w* and *I* matched to it, while *G* and σ remain unchanged. By adjustment of the parameters, a 53% improvement was obtained in the correlation between simulated FC and empirical FC, as estimated from a comparison of the empirical SC with the original model.

**Figure 1:**
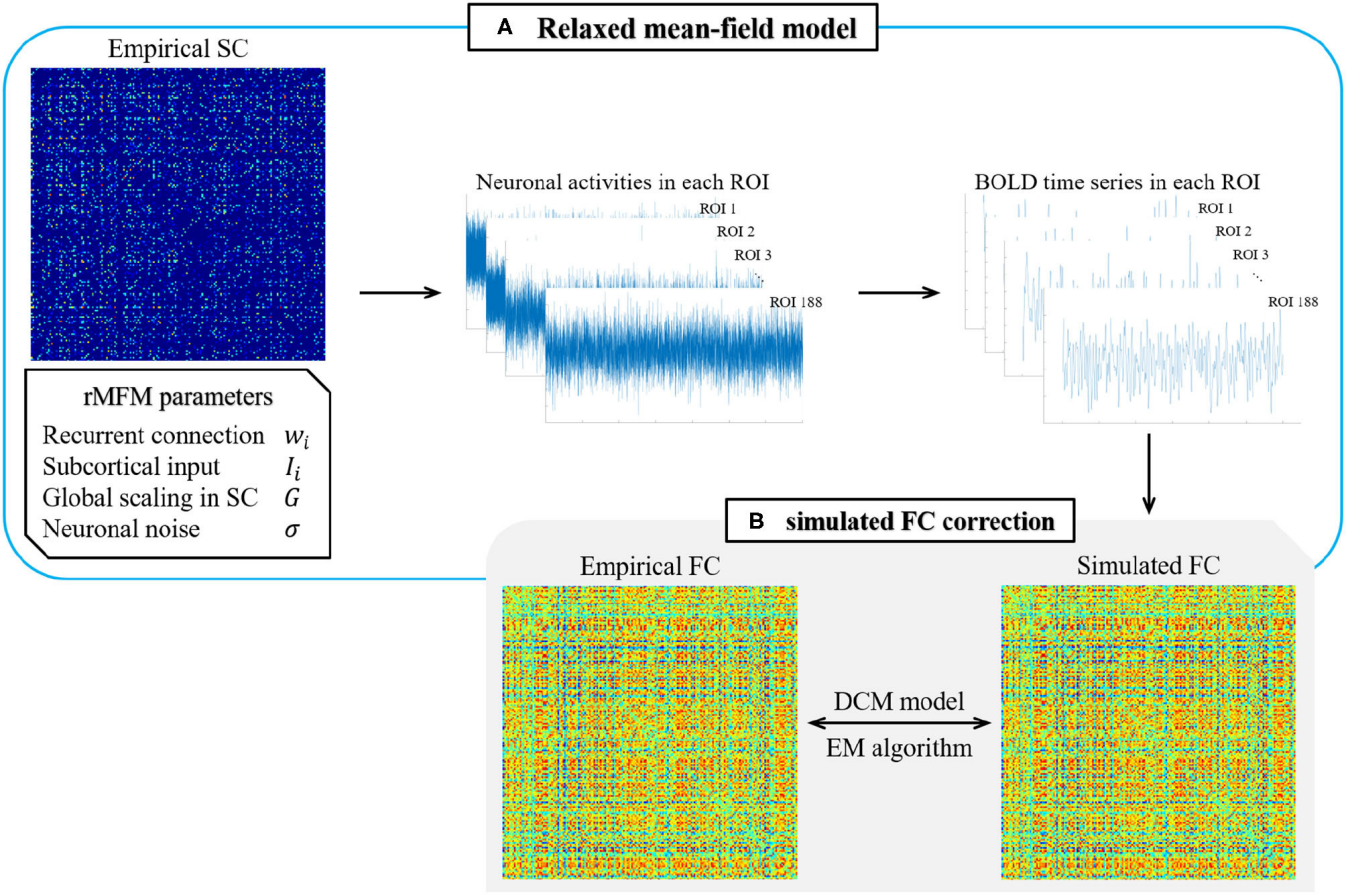
Overview of rMFM & simulated FC correction. **(A)** Relaxed mean-field model. The empirical SC and initial parameters are given to the relaxed mean-field model to obtain the neuronal activities in each ROI and then input the neuronal activity into the hemodynamic model to obtain the BOLD time series. Calculated the correlation of the BOLD time series to obtain the simulated FC. **(B)** Simulated FC correction. Using the maximum expectation algorithm in the dynamic causal model to correct the simulated FC.

Therefore, we used the rMFM model to predict the neuronal activity in each ROI. As shown in Figure 1, we took the SC state of each ROI as the model input while given a set of initial parameters to simulate the neuronal activity of each ROI. Then, the BOLD time series of each ROI were simulated using the Balloon–Windkessel hemodynamic model, and the Pearson correlation coefficients of the BOLD time series of each ROI were calculated to give the simulated FC. Finally, we used the empirical FC to correct the model parameters and substituted the corrected parameters into the model until the error accuracy met the required level or the simulation time reached a stipulated maximum value.

### 2.3. Parameter Initialization Analysis

Although the rMFM model improves the correlation between simulated and empirical FC, neither Deco et al. (2013) nor Wang et al. (2019) specified the impact of initial parameters on the final fitting correlation in establishing the model. In general, different initial parameters will lead to different results from a model, and we believe that the rMFM model is no exception in this respect. We therefore designed a numerical simulation to examine this issue.

First, we averaged the empirical SC and FC of all 196 samples to get the group average SC and FC. These two groups of average connectivity matrices were used only to explore the initial parameter sensitivity of the rMFM model, and were not used as a reference in the later evolution analysis experiments.

Next, we used the control variable method to study the influence of different parameter initializations on the results. For *w*_*i*_ and *I*_*i*_ taking uniform values in steps of 0.1 within the range [0.1, 0.9], 81 groups of initial parameters were obtained. After model fitting, the correlation between simulated and empirical FC was recorded, and we obtained the correlation surface in the space of initial parameters. On this basis, we took more precise steps for the initial parameter interval with high correlation to get more accurate results. Although this method is simple, it confirmed our belief regarding the sensitivity of the results to the initial values of the parameters (see section 4.1 for specific results).

### 2.4. Estimation of Model Parameters

We simulated all 196 samples and obtained 378 parameters (188 recurrent connection strengths *w*_*i*_, 188 subcortical input strengths *I*_*i*_, a global scaling factor *G*, and a noise coefficient σ).

After we had obtained the first simulated FC using the initial parameters, we optimized the parameters by using the maximum expectation algorithm in dynamic causal modeling (Friston et al., 2003) (see Wang et al., 2019 for the detailed steps of the algorithm). We optimized each sample for 500 iterations and selected the one with the highest Pearson correlation between simulated and empirical FC as the final parameter of the sample.

After all 196 sample parameters were fitted, we took out the young group and the old group, performed a two-sample *t*-test for each parameter for each ROI, and marked the ROIs with significant difference (*p* < 0.05, FDR corrected). We will present our results in detail in section 3.1.

Next, we drew scatter plots of parameter values with age for all ROIs, and fitted these values with first- and second-order polynomials. Through polynomial fitting, we could see how each parameter of the ROI changes with age. We will present these results in detail in section 3.2.

## 3. Results

### 3.1. Comparison of Model Parameters

**Figure 2:**
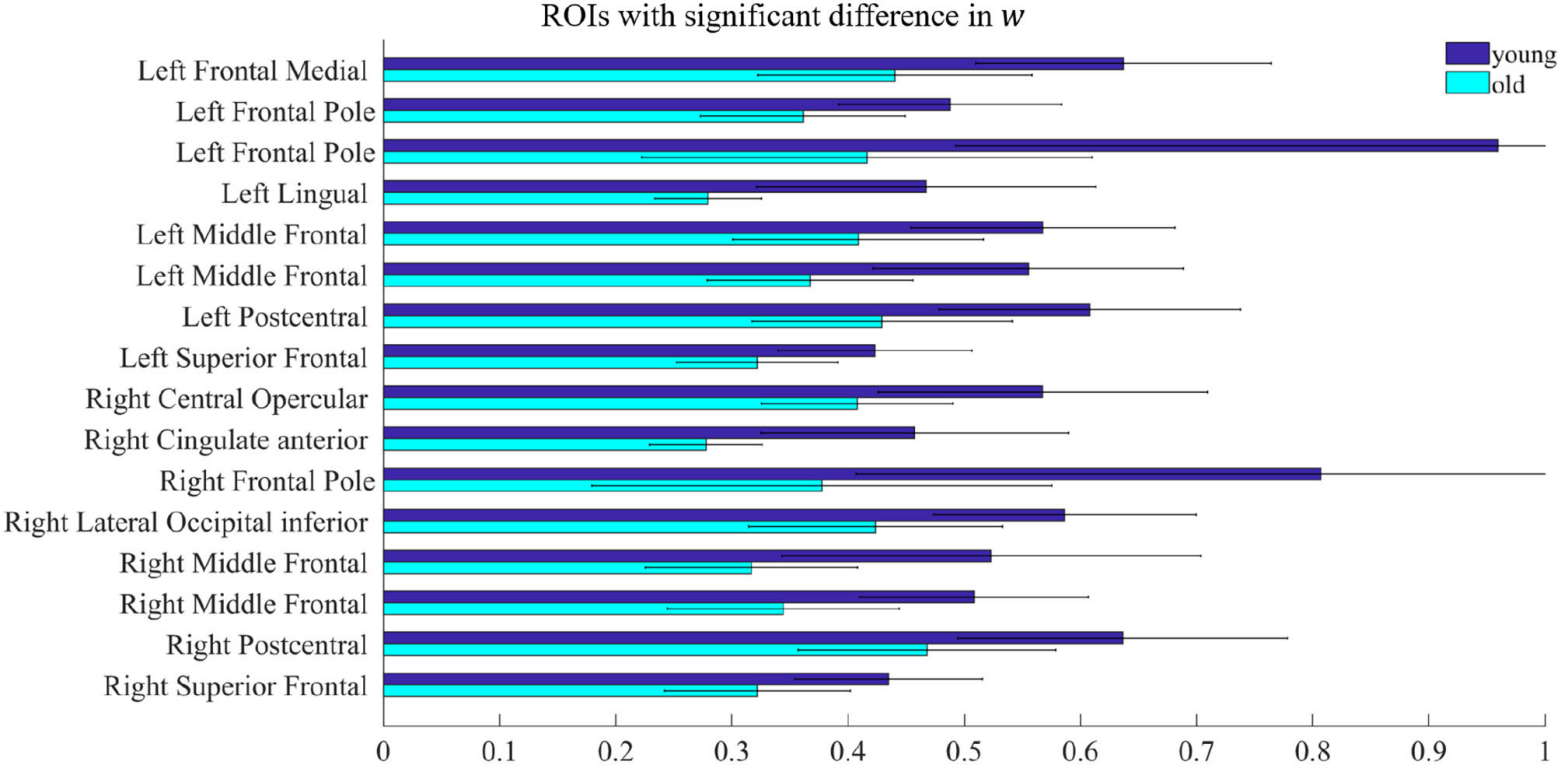
ROIs with significant difference in *w*. Each pair of bars represents the mean value of the parameter *w* in the same ROI for the young group (dark blue) and the old group (light blue). The lines on the bars indicate the standard deviation.

All model fitting results were obtained by running the simulations on MATLAB R2020a (MathWorks Inc., Natick, MA, USA). By comparing the parameters *w* and *I* of the rMFM model, we studied the micro brain characteristics of the young group and the old group. Here, we used the two-sample *t*-test to judge whether each ROI showed significant differences between the two groups. We found ROIs with significant differences in recurrent connection strength *w*, as shown in Figure 2, but we did not find ROIs with significant differences in subcortical input strength *I*. Because of the large number of ROIs, for clarity, only ROIs with significant differences are shown in Figure 2 (see Table 2 for statistical information).

**Table 2:** ROIs with significant difference in *w*.

**ROI names full (abbrev)**	**Young group mean (±std)**	**Old group mean (±std)**	***p***_***corrected***_
Left Frontal Medial (LFMC)	0.64 (± 0.13)	0.44 (± 0.12)	0.0336
Left Frontal Pole (LFP)	0.49 (± 0.10)	0.36 (± 0.09)	0.0395
Left Frontal Pole (LFP)	0.96 (± 0.47)	0.42 (± 0.19)	0.0363
Left Lingual (LLG)	0.47 (± 0.15)	0.28 (± 0.05)	0.0252
Left Middle Frontal (LMFG)	0.57 (± 0.11)	0.41 (± 0.11)	0.0376
Left Middle Frontal (LMFG)	0.56 (± 0.13)	0.37 (± 0.09)	0.0291
Left Postcentral (LPG)	0.61 (± 0.13)	0.43 (± 0.11)	0.0376
Left Superior Frontal (LSFG)	0.42 (± 0.08)	0.32 (± 0.07)	0.0460
Right Central Opercular (RCOC)	0.57 (± 0.14)	0.41 (± 0.08)	0.0470
Right Cingulate anterior (RCGad)	0.46 (± 0.13)	0.28 (± 0.05)	0.0311
Right Frontal Pole (RFP)	0.81 (± 0.40)	0.38 (± 0.20)	0.0460
Right Lateral Occipital inferior (RLOCid)	0.59 (± 0.11)	0.42 (± 0.11)	0.0399
Right Middle Frontal (RMFG)	0.52 (± 0.18)	0.32 (± 0.09)	0.0401
Right Middle Frontal (RMFG)	0.51 (± 0.10)	0.34 (± 0.10)	0.0382
Right Postcentral (RPG)	0.64 (± 0.14)	0.47 (± 0.11)	0.0444
Right Superior Frontal (RSFG)	0.43 (± 0.08)	0.32 (± 0.08)	0.0391

In Figure 2, there were 16 ROIs with significant differences in the parameter *w* (*p* < 0.05, FDR corrected), accounting for 8.5% of the total ROIs. These ROIs are mainly concentrated in the bilateral frontal pole, the bilateral superior frontal, bilateral middle frontal, bilateral postcentral, and a few areas of the occipital. We can also see in Figure 2 that for recurrent connection strength *w*, the average value of the old group is significantly weaker than that of the young group in the above ROIs, and shows high consistency. For example, in one left frontal pole, the mean parameter value in the young group is above 0.9, while in the old group it is less than 0.5. The same is the case for one right frontal pole, which is above 0.8 in the young group and only about 0.4 in the old group.

For subcortical input strength *I*, we did not find ROIs with significant differences between the young and the old group. However, this is only the result of the two-sample *t*-test and does not mean that there is no change in subcortical input strength with age. We will show the reason for this in the next subsection.

**Figure 3:**
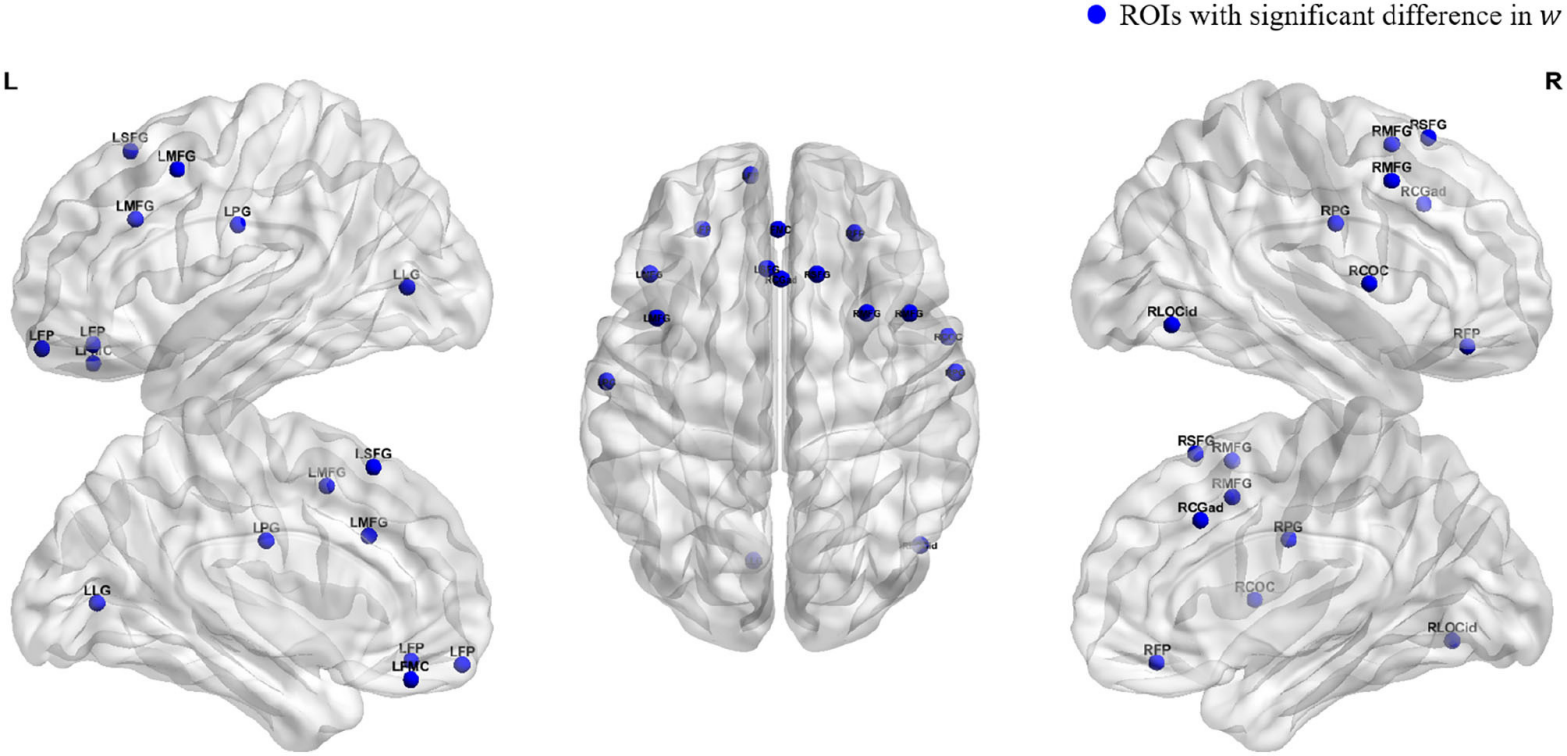
Spatial locations of ROIs. Blue dots indicate the locations of the ROIs which the parameter *w* is significant.

To obtain an intuitive view of the ROIs with significant differences, we used the BrainNet toolbox (Xia et al., 2013) to map the spatial positions of the above regions in the brain, as shown in Figure 3. We can see that the ROIs with significant differences between the young group and the old group are mainly concentrated in the frontal pole, frontal and central regions, and a few occipital regions.

### 3.2. Trends of Parameter Changes

**Figure 4:**
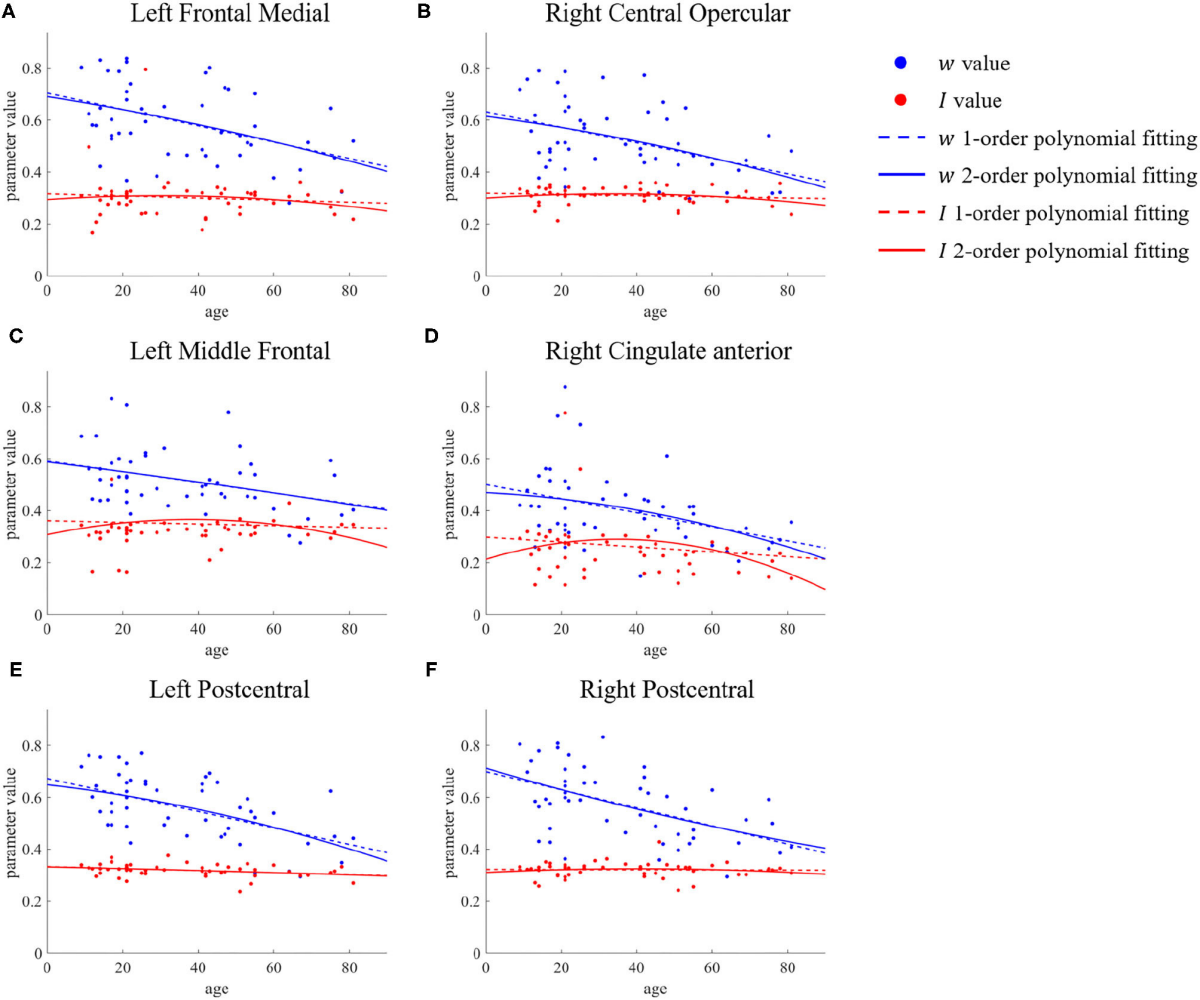
ROIs with no significant difference in first- and second-order polynomial fits of recurrent connection strength *w*. The points represents the parameter values, the dashed lines are the first-order polynomial fits, and the solid lines are the second-order polynomial fits. Blue indicates *w* and red indicates *I*. **(A)** Left Frontal Medial. **(B)** Right Central Opercular. **(C)** Left Middle Frontal. **(D)** Right Cingulate anterior. **(E,F)** Bilateral Postcentral.

Following the results described in the previous subsection, we carried out a further statistical analysis on regions such as the frontal pole, superior frontal, middle frontal, postcentral, and occipital. Taking the left frontal medial as an example, we extracted the values of the parameters *w* and *I* of all samples, and then drew scatter plots to observe the changes with aging. Finally, we used first- and second-order polynomials to fit these parameters, giving the results shown in Figure 4A. As well as the left frontal medial, the results for the left middle frontal, right central opercular, right cingulate anterior, and bilateral postcentral are also shown in Figure 4. We can see that for the recurrent connection strength *w*, there are significant differences between the young group and the old group. When all samples are combined, the second-order polynomial used for fitting almost overlaps with the first-order polynomial, which means that the recurrent connection strength decreases linearly and slowly with age.

**Figure 5:**
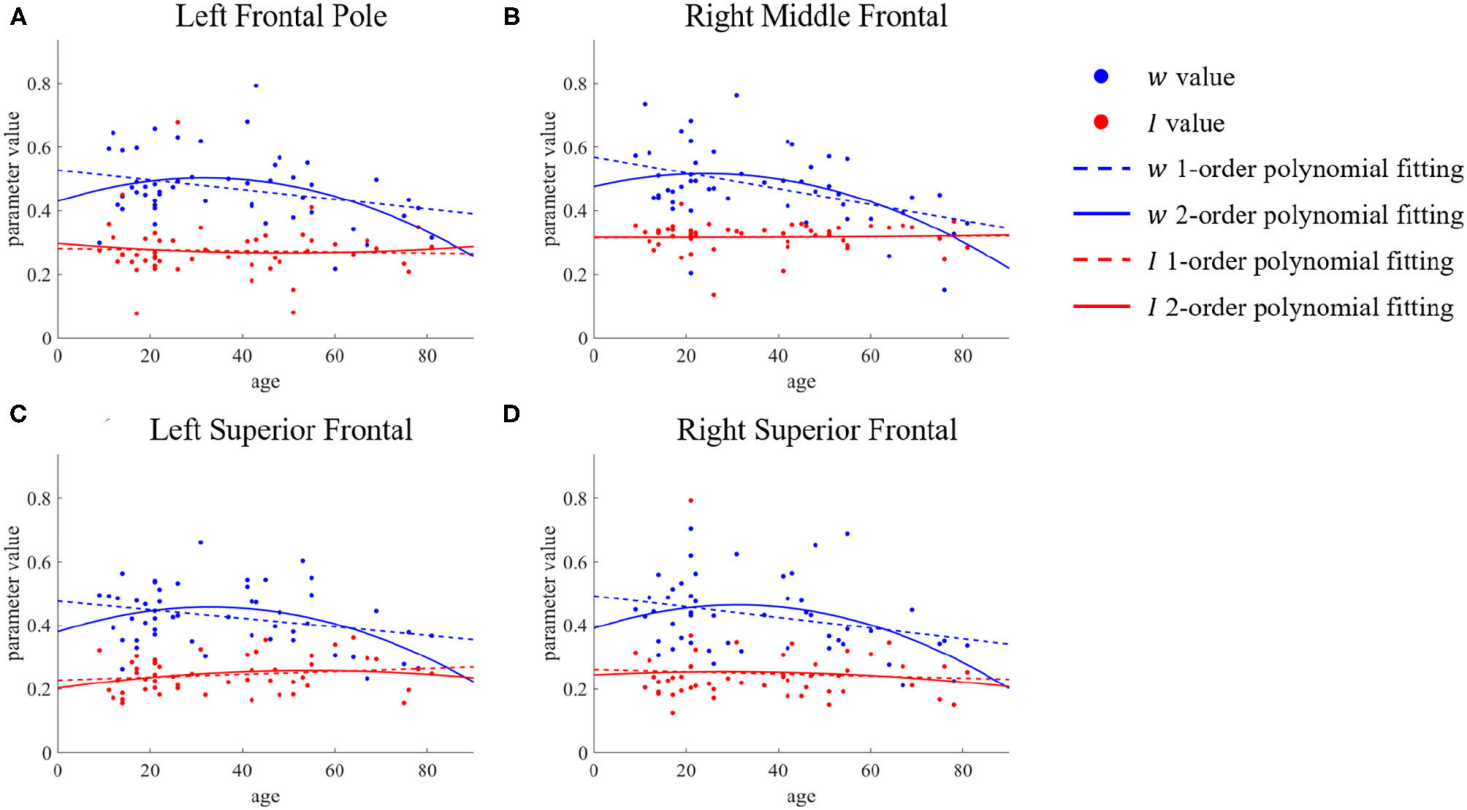
ROIs with differences in first- and second-order polynomial fits of recurrent connection strength *w*. The points represent the parameter values, the dashed lines are the first-order polynomial fits, and the solid lines are the second-order polynomial fits. Blue indicates *w* and red indicates *I*. **(A)** Left Frontal Pole. **(B)** Right Middle Frontal. **(C,D)** Bilateral Superior Frontal.

However, the situation is different for the left frontal pole, right middle temporal, and bilateral superior frontal. We present the parameter changes of these ROIs in Figure 5. Figure 5A shows the results for the left frontal pole, while Figure 5B shows the results for the right middle frontal, and Figures 5C,D for the bilateral superior frontal. We can see that although there is a significant difference in the value of the parameter *w* between the young group and the old group, the value for ages between 30 and 60 is almost the same as in the young group. The time at which these ROIs change in recurrent connection strength is from middle age to old age, but there is no significant change before that. The second-order polynomial fit presents another unique state, an inverted U-shape. With increasing age, this micro brain characteristic first gradually increases until it becomes stable in middle age, after which it decreases.

**Figure 6:**
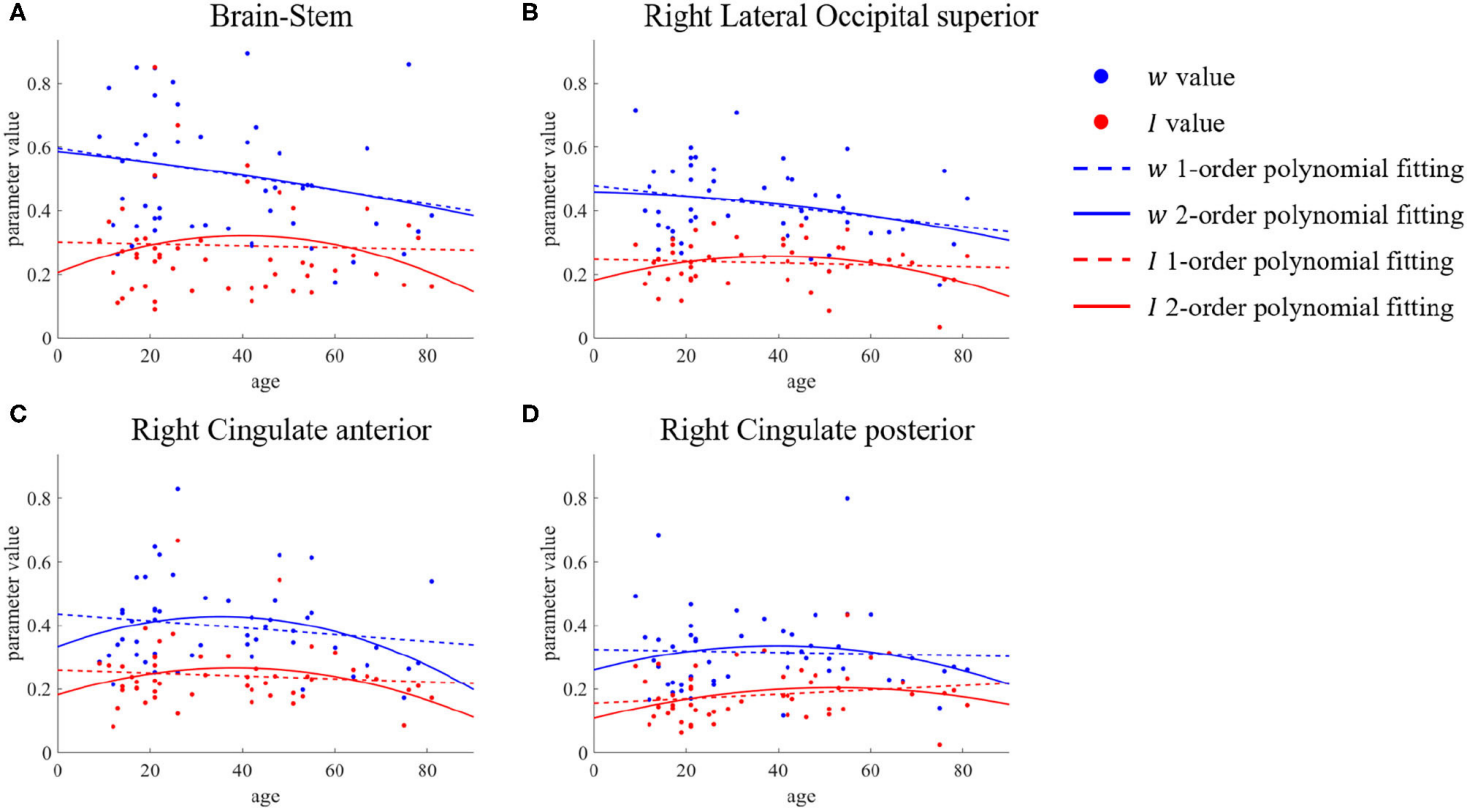
ROIs with differences in first- and second-order polynomial fits of subcortical input strength *I*. The points represent the parameter values, the dashed lines are the first-order polynomial fits, and the solid lines are the second-order polynomial fits. Blue indicates *w* and red indicates *I*. **(A)** Brain-Stem. **(B)** Right Lateral Occipital superior. **(C,D)** Right Cingulate anterior & posterior.

The above is an analysis of the trend of recurrent connection strength *w* with age. In section 3.1, we mentioned that for subcortical input strength *I*, we did not find ROIs that showed significant differences between the young group and the old group by the two-sample *t*-test, but this does not mean that this microscopic brain character did not change with age. The second-order polynomial fit of parameter *I* was similarly inverted U-shaped in these brain regions of the brain-stem, right lateral occipital superior, and right cingulate shown in Figure 6. There was an increase from young to middle age and a decrease from middle to old age, so that the difference in the parameter *I* between the young group and the old group could not be tested using only a two-sample *t*-test.

## 4. Discussion

We have used the rMFM model to study the effect of aging on micro brain characteristics under natural conditions. We have found that aging will lessen the recurrent connection strength in most ROIs, including frontal pole, superior frontal, middle frontal, postcentral, central opercular, and cingulate anterior. These differences in micro brain characteristics show different trends of change with age.

### 4.1. Influence of Initial Parameters

**Figure 7:**
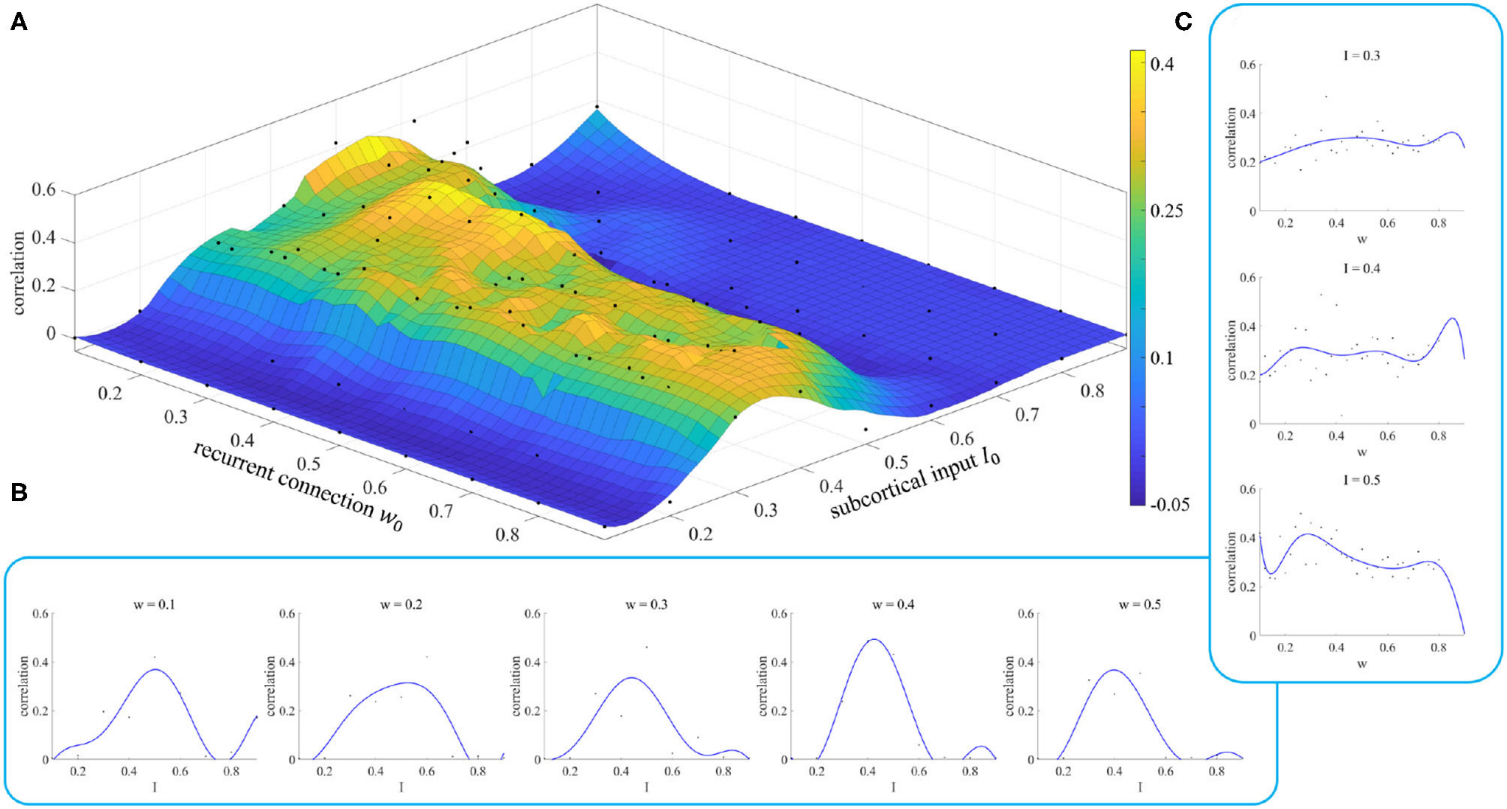
Simulation results of parameter initialization. **(A)** Each point represents the correlation result corresponding to a pair of parameters (*w, I*). **(B)** Variation of the correlation with the parameter *I* for given values of the parameter *w*. **(C)** Variation of the correlation with the parameter *w* for given values of the parameter *I*.

In section 2.3, we noted that different parameter initializations will lead to different results, and we designed an experiment to prove this conclusion. The results of this experiment are shown in Figure 7.

Figure 7A shows the fitting results of the model when we took different values of the initial parameters. We can see when the parameter *I* is less than 0.2 or greater than 0.6, the correlation between simulated and empirical FC is close to 0, which means that we cannot get effective results. The appropriate initial range for the parameter *I* is [0.3, 0.5]. The initial value of the parameter *w* does not have such a significant effect. However, we can still see when the initial value of *I* is in the range [0.3, 0.5], small initial values of the parameter *w* give a better correlation than for large ones.

Figures 7B,C show the changes in fitting results as one parameter varies while the other is fixed. These curves enable us to select the initial value of the parameter more easily. Neither the dependence on *w* nor that on *I* shows the characteristics of a smooth curve. This is because there is a parameter σ, called neuronal noise, that arises in the process of rMFM model fitting, and the randomness of this parameter leads to jitter in a single simulation curve. If the simulation was repeated a number of times and the average value of the results was taken, this reduced the jitter and gave a smoother result.

### 4.2. Micro Brain Characteristics With Aging

For the recurrent connection strength *w*, the brain regions that showed significant differences between the young group and the old group were mostly distributed in the frontal, including left frontal medial, frontal pole, superior frontal, and middle frontal. The surface area of the frontal pole is negatively correlated with visuospatial working memory ability (Zacharopoulos et al., 2020) and plays an important role in controlling and maintaining information related to its behavior (Arai et al., 2016). And it is the frontal region that is closely related to cognitive ability. Damage to this region will lead to impairment of cognition, extraction of vocabulary, and other functions (Wierenga et al., 2008; Wang et al., 2010; Goh et al., 2013).

Other regions, such as right lateral occipital inferior, postcentral, right central opercular, and right cingulate anterior, similarly exhibited intergroup differences. The right occipital pole is part of the occipital cortex. The fractional anisotropy (FA) of its connecting bundle with the thalamus decreases significantly with age, which affects visual short-term memory ability (Menegaux et al., 2019, 2020). Some experiments have shown that with an increase in working memory load, young people can activate the default mode network (DMN) region faster than old people. Meanwhile, the neuroregulatory ability of young people is significantly higher (Qin and Basak, 2020).

Corticobasal syndrome (CBS) is a progressive movement disorder characterized by akinetic–rigid parkinsonism and a varying combination of motor and nonmotor symptoms. It is typically asymmetric and affects a single body region, especially the upper limbs. Upadhyay et al. (2016) found that the cortical thickness (CTh) of the precentral gyrus of patients with CBS was significantly lower than that of healthy people, and the CTh asymmetry of the postcentral gyrus was negatively correlated with the duration of the disease. Multiple sclerosis is a chronic neurodegenerative disease associated with somatosensory abnormalities and decreased stability (Moore et al., 2012). The postcentral gyrus is the central region of the somatosensory network (Tomasi and Volkow, 2011), and for patients with multiple sclerosis with somatosensory disorders, the functional connection of the postcentral gyrus is significantly weaker than in healthy groups (Fu et al., 2019). As a result, the weakening of the postcentral will affect the human ability to behave.

The cingulo-opercular network is closely related to the speed of visual processing. Through a study of this network, Ruiz-Rizzo et al. (2019) found that the internal functional connection (iFC) of the right anterior paracingulate and bilateral middle paracingulate decreased significantly with age, leading to a decrease in the speed of visual processing in the old group. A decrease in visual processing speed will reduce alertness, and thus maintaining alertness is also an important function of the cingulo-opercular network (Coste and Kleinschmidt, 2016; Haupt et al., 2019).

### 4.3. Limitations

Our study did have some limitations. First, the number of samples was small, so we were not able to group more delicately. Given a sufficient number of samples with uniform age distribution, the accuracy of age-related changes in micro brain characteristics could be improved. Second, we grouped according to age, and obtained our results by comparing parameters between young and old groups, and the results could easily have been affected by individual differences, i.e., outliers. If we can obtain long-life-cycle data of several samples from youth to old age, we may be able to draw further conclusions. Then, the dataset we used did not provide the original neuroscience imaging data, only the constructed connectivity matrix. Therefore, we have no way to do simulation evaluation, such as the number of ROIs, different atlases, etc. Finally, we did not carry out a theoretical derivation of the parameter initialization analysis, but just used a parameter fitting based on the ROI divided according to the Craddock 200 atlas, and we did not corroborate this by substituting another data set or atlas. Therefore, we propose that other researchers use the rMFM model to initialize and analyze their data to get initial values of the parameters.

## 5. Conclusion

In this article, we used the rMFM model to study the effect of aging on micro brain characteristics. First, we analyzed the initial parameter sensitivity of the rMFM model and selected an initial parameter range. We then identified the ROIs with significant differences in recurrent connection strength between young and old groups, mainly in the frontal, postcentral, central opercular, and cingulate anterior, as well as ROIs such as right lateral occipital inferior and left lingual. Further, we studied the trends of change of these ROIs with age. For some ROIs, such as the frontal medial, left middle frontal, and postcentral, the decrease in recurrent connection strength was found to follow a roughly linear trend with age. However, for other ROIs, such as left frontal pole, right middle frontal, and right cingulate, the decrease in recurrent connection strength or subcortical input strength was found to be manifested mainly in the middle-aged to elderly. The weakening of SC and FC caused by aging is irreversible, but means to effectively delay brain aging are still worthy of more research.

## Data Availability Statement

The Nathan Kline Institute (NKI)/Rockland Sample are publicly available (http://fcon_1000.projects.nitrc.org/indi/pro/nki.html). The codes for the rMFM model used in the paper are adopted from https://github.com/ThomasYeoLab/CBIG/tree/master/stable_projects/fMRI_dynamics/Wang2018_MFMem.

## Author Contributions

ST, YZheng, and KZ contributed to the conception and design of the study. ZZ provided the equipment for the experimental platform. KZ wrote the first draft of the manuscript. ST, YZheng, YY, YZhen, and KZ wrote various parts of the manuscript. All authors contributed to manuscript revision, and, approved the submitted version.

## Funding

This work was supported by the Program of the National Natural Science Foundation of China (Grant Nos. 42050105, 11871004, and 11922102) and the National Key Research and Development Program of China (Grant No. 2018AAA0101100).

## Conflict of Interest

The authors declare that the research was conducted in the absence of any commercial or financial relationships that could be construed as a potential conflict of interest.

## Publisher's Note

All claims expressed in this article are solely those of the authors and do not necessarily represent those of their affiliated organizations, or those of the publisher, the editors and the reviewers. Any product that may be evaluated in this article, or claim that may be made by its manufacturer, is not guaranteed or endorsed by the publisher.
